# Treatment planning for lung cancer reirradiation accounting for previously delivered dose^☆^

**DOI:** 10.1016/j.phro.2026.100912

**Published:** 2026-02-06

**Authors:** David P. Walton, Christopher Thompson, Dominic Lowe, Christopher J.H. Pagett, John Lilley, Stina Svensson, Kjell Eriksson, Rasmus Bokrantz, Jakob Ödén, Louise Murray, Mark Teo, Ane Appelt

**Affiliations:** a Department of Radiotherapy Physics, Barts Health NHS Trust, London, UK; b Department of Medical Physics, Leeds Cancer Centre, Leeds Teaching Hospitals NHS Trust, Leeds, UK; c Department of Clinical Oncology, Leeds Cancer Centre, Leeds teaching Hospitals NHS Trust, Leeds, UK; d RaySearch Laboratories, Stockholm, Sweden; e Leeds Institute of Medical Research at St James’s, University of Leeds, Leeds, UK

**Keywords:** Reirradiation, Treatment planning, Lung cancer, Dose accumulation, Equieffective dose

## Abstract

Reirradiation is increasingly used for recurrent and new primary cancers, but dedicated planning tools are limited. We integrated deformable image registration and radiobiologically consistent dose accumulation into plan optimisation to improve plan quality and robustness, applying this approach to six lung cancer cases and comparing it with a manual, maximum point-dose method.

The integrated automated approach delivered higher target doses within organ-at-risk limits, maintained robustness, and was qualitatively preferable in blinded comparison. It produced clinically acceptable dose distributions for 5/6 patients, versus 3/6 with the manual method, suggesting enhanced and potentially more efficient reirradiation planning.

## Highlights

• Lung reirradiation planning was automated using cumulative equieffective dose.
• For six cases, automated planning avoided 96 manual dose calculations and checks.
• Change of PTV D_99%_ ranged from −2.1 Gy to 16.7 Gy (median 2.1 Gy).
• Blinded clinician review picked equieffective dose optimisation in 5/6 cases.

## Graphical abstract

**Figure:**
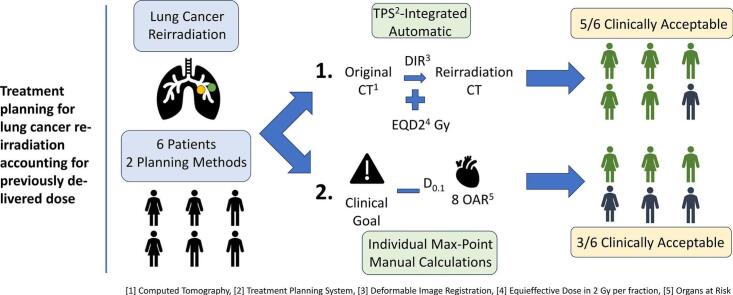

## 1 Introduction

Reirradiation, administering new courses of radiotherapy to previously irradiated regions, is increasingly used for recurrent or new primary cancer [1], [2], [3], [4]. The lack of integrated reirradiation tools in commercial treatment planning systems (TPS) hinders adoption of radiobiologically corrected (equieffective [5]) 3D dose distributions for cumulative dose assessment and planning, a key gap in treatment planning software [6].

Reirradiation planning must spatially incorporate the previous dose distribution, requiring iterative cumulative equieffective dose evaluation during optimisation. We previously developed this functionality in a commercial TPS, demonstrating its applicability in pelvic and brain reirradiation [7], [8]. Our approach uses dedicated cost functions that optimise cumulative equieffective dose in 2 Gy fractions (EQD2Gy) voxel-by-voxel [9], [10].

New or recurrent lung tumours after initial (chemo)radiation are increasingly managed with reirradiation [2], [9]. A recent international Delphi exercise provided guidance for thoracic cumulative constraints [11]. Previous studies demonstrated the value of deformable image registration (DIR) and EQD2Gy cumulative dose evaluation in lung reirradiation [12], [13], and explored workarounds for plan optimisation [14], [15]. However, no prior work has addressed integrated planning solutions for thoracic reirradiation, fully accounting for cumulative equieffective dose.

Addressing this challenge, we demonstrated the feasibility and clinical utility of such an approach, verified the clinical acceptability of plans and compared them with maximum point-dose methods in blinded evaluation. Additionally, we quantified patient- and organ-specific dose mapping uncertainties, assessing plan robustness.

## 2 Materials and methods

### 2.1 Cohort and data

The study was based on data from six patients who had previously undergone primary lung cancer radiotherapy followed by reirradiation at Leeds Teaching Hospitals NHS Trust (LTHT). All had limited anatomical changes between courses (e.g., no major surgery). The first (i.e. previous) treatment used stereotactic ablative radiotherapy (SABR); all reirradiation treatments used non-SABR techniques.

Ethics approval for retrospective data was granted by The Leeds Cancer Centre Computer Aided Theragnostics (LeedsCAT) research database (Research Ethics Committee reference 19/YH/0300). All work was conducted in RayStation11A (RaySearch Laboratories AB, Sweden; research version 11.0.110).

Clinical computed tomography (CT) scans, structure sets, and dose distributions were imported into a research version of RayStation11A. A consultant clinical oncologist reviewed organ-at-risk (OAR) delineations against a standard structure set; missing or substandard OARs were added or edited on both imaging datasets. Sup. Table S1 provides a breakdown of patient and target characteristics.

### 2.2 Image registration

Spine and bony anatomy were contoured using a Hounsfield unit (HU) threshold algorithm to guide registration. Rigid image registration (RIR) was performed with the reirradiation CT as reference, followed by a DIR using ANACONDA (anatomically constrained deformation algorithm) [16]. Bone contours and lungs served as controlling structures; lungs were the focus region of interest.

A consultant clinical oncologist qualitatively evaluated whether the DIR accurately reflected changes in patient anatomy. A side-by-side comparison with the RIR was conducted, the vector field was visualised to identify areas requiring significant deformation, and key OAR were studied individually to assess the plausibility of the structure propagation. Jacobian calculations ensured all deformations were physical.

Unacceptable registrations were repeated using additional OARs. Approved deformation vector fields were then used to map the previous dose onto the reirradiation CT. DIR performance for each OAR was estimated using the mean distance to agreement (MDA), providing an assessment limited to the OAR boundary region.

### 2.3 Treatment planning

To standardise the clinical goals for assessing cumulative dose distributions, all reirradiation plans used 60 Gy in 30 fractions. Full details of the planning approach can be found in Supplementary Material A. Plans were research-only and created by one physicist.

We applied two sets of clinical goals: i) *de novo* lung radiotherapy goals for the reirradiation plan only, and ii) combined EQD2Gy goals developed with a consultant clinical oncologist and informed by the literature (see Sup. Table S2, which also lists α/β values). No combined lung goal was set due to a lack of consensus. Clinical goals included optimal and mandatory criteria and informed initial optimisation objectives.

Two planning approaches were compared. The first used STRIDeR (Support Tool for ReIrradiation Decisions guided by Radiobiology). This employed OAR optimisation functions that incorporated voxel-wise cumulative EQD2Gy during optimisation [10], using the mapped previous dose and with OAR-specific α/β values (Sup. Table S2). Cumulative EQD2Gy goals could be iteratively evaluated during optimisation (see [7] for details) [17].

The second was a commonly used point-dose based manual approach, representative of practice in some centres. The maximum dose to 0.1 cm^3^ was extracted from the previous plan for each of the eight OARs with combined clinical goals, converted into EQD2Gy, and subtracted from the combined clinical goals to derive patient-specific residual limits. Each required an independent check. Residuals, rescaled to 30-fractions, were manually entered for TPS optimisation objectives.

Both methods were optimised by reducing target coverage until mandatory OAR clinical goals were achieved. Optimal clinical goals were pursued when achievable without compromising target coverage.

### 2.4 Cumulative dose Estimation

Dose distributions from previous treatments were deformed onto reirradiation planning CTs, where each OAR was assigned an α/β value (Sup. Table S2). If a dose voxel was assigned to multiple OARs, a priority list from lowest to highest α/β value was used. Dose voxels within the external contour not designated as OARs were assigned an α/β value of 3 Gy. Both dose distributions were subsequently rescaled to EQD2Gy and summed voxel-by-voxel to evaluate the cumulative doses on the reirradiation CT. EQD2 metrics were subsequently calculated using the full set of voxels assigned to an OAR, as per the RayStation patient model.

Robust cumulative dose estimates accounted for geometric uncertainties in DIR and dose mapping. For each OAR that had cumulative clinical goals, each OAR mapped dose voxel was resampled and replaced by the maximum dose inside a sphere generated on the unmapped previous dose and centred at the source position of the mapped dose voxel. The spherical resampling kernel radius used was the organ- and patient-specific MDA to approximate the uncertainty in image registration [8] (Sup. Table S3). Radiobiological uncertainty was addressed by assigning each OAR a published α/β range (Sup. Table S2), varying α/β in integer steps, recalculating the combined equieffective dose, and recording the largest estimate.

For each voxel, the maximum from geometric and radiobiological resampling was used for the robust dose distribution. Uncertainties were quantified separately for OAR with combined clinical goals and compared per organ to identify the dominant source.

### 2.5 Reirradiation plan evaluation

Plans were deemed clinically acceptable if all mandatory OAR and target clinical goals were achieved. Target coverage was assessed by comparing the dose to 99% of the planning target volume (PTV) (D_99%_) between STRIDeR and manual plans.

A consultant clinical oncologist, blinded to planning method and case order, evaluated each plan on a five-point Likert scale: 5 = clinically acceptable, no changes required; 4 = clinically acceptable with potential for minor optimisation; 3 = clinically acceptable but failed optimal OAR constraints; 2 = clinically unacceptable, replan required; 1 = unachievable with the current prescription.

The oncologist also provided a blinded comparative preference for each patient: strong, weak, or no preference, between the two plans.

## 3 Results

All cases achieved a clinically acceptable DIR; two initial unacceptable registrations were corrected by adding the proximal bronchial tree in one case and the pericardium in the other as additional controlling structures. Most OARs were within 2 mm MDA, except the brachial plexus, median 4.9 mm (range 4.3 mm to 5.3 mm) (Sup. Table S3).

**Fig. 1:**
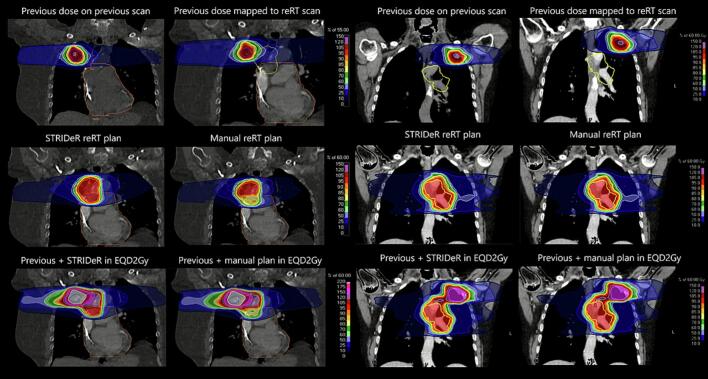
Previous and reirradiation images and treatment plans for Patient 3 (left) and Patient 1 (right). Patient 3: The previous plan delivered 55 Gy in 5 fractions to the planning target volume (PTV) (upper left, blue contour, 49.0 cm^3^). The previous dose was mapped onto the reirradiation (reRT) CT (upper right). The Support Tool for ReIrradiation Decisions guided by Radiobiology (STRIDeR) approach permits coverage of the inferior section of the reRT PTV (centre left) compared with the manual approach (centre right). Lower images show the cumulative dose in EQD2Gy for STRIDeR (left) and manual (right) plans. Orange contour: pericardium; yellow contour: reRT PTV (56.7 cm^3^). Patient 1: The previous plan delivered 60 Gy in 8 fractions to the PTV, left upper lobe (36.7 cm^3^). The second treatment was in the left lower lobe, PTV volume (275.8 cm^3^). Both planning methods produced very similar plans, demonstrating that in some cases there is little dosimetric benefit; however the STRIDeR method was considered less burdensome and more automated by the planner.

Five STRIDeR plans were deemed clinically acceptable, compared with three manual plans (Table 1). Differences were due to OAR with combined clinical goals overlapping the PTV. STRIDeR accounted for spatial distribution of previous dose, covering PTV that the manual method assumed overdose. Fig. 1 illustrates where STRIDeR achieved better target coverage near the pericardium by accounting for previous dose distributions. The change in PTV D_99%_ from manual plans to STRIDeR plans ranged from –2.1 Gy to 16.7 Gy (median 2.1 Gy), see Table 1.

**Table 1:** Quantitative and qualitative planning results for Support Tool for ReIrradiation Decisions guided by Radiobiology (STRIDeR) approach and manual approaches. Numbers represent number of goals that passed for the plan in question. Plans were considered clinically acceptable and passed if they achieved all target clinical goals and mandatory clinical goals for organs at risk (OAR). Note that patient two had no contralateral lung clinical goals as there were targets in both lungs.

Patient		1		2		3		4		5		6	
Quantitative		STRIDeR	Manual	STRIDeR	Manual	STRIDeR	Manual	STRIDeR	Manual	STRIDeR	Manual	STRIDeR	Manual
	Overall	Pass	Pass	Pass	Fail	Pass	Fail	Pass	Pass	Fail	Fail	Pass	Pass
	Optimal OAR clinical goals	10	10	7	7	9	9	9	9	9	9	10	10
	Mandatory OAR clinical goals	17	17	17	17	17	17	17	17	17	17	17	17
	Target coverage clinical goals	8	8	8	6	8	5	8	8	4	4	8	8
	D_99%_ (Gy)	57.7	57.5	58.1	41.4	57.4	49.7	56.2	57.0	13.4	9.4	56.2	58.3
Qualitative													
	Likert Scale Score	5	5	4	1	4	1	4	4	1	1	5	5
	Clinical Preference	Weak STRIDeR		Strong STRIDeR		Strong STRIDeR		Weak STRIDeR		Weak STRIDeR		No Preference

Clinician review preferred STRIDeR in five of the six cases; in the remaining case, where the reirradiation PTV lay outside the superior-inferior plane of the previous treatment, no preference was expressed. When both planning methods achieved clinically acceptable plans, STRIDeR was favoured for dose coverage around the target.

Robust resampling showed most OARs met clinical goals, except for the brachial plexus, which exceeded tolerance in 50% of patients for both methods (Sup. Table S4). In one case, the STRIDeR plan exceeded mandatory proximal bronchial tree constraint by 1.8 EQD2Gy (α/β = 2 Gy), whereas the manual plan remained within tolerance. For the trachea, one manual plan exceeded tolerance by 1.7 EQD2Gy (α/β = 2 Gy), while the STRIDeR plan met the constraint.

Uncertainty contributions varied by organ. Dose mapping dominated for brachial plexus and pericardium, (median differences 10.7 Gy and 0.6 Gy) (Sup. Table S5), whilst α/β uncertainty predominated for other OARs, largest in the spinal canal (1.8 Gy) and trachea (0.9 Gy). The MDA metric did not detect dose mapping uncertainty in the spinal canal in any case.

## 4 Discussion

This study demonstrated the feasibility of full equieffective cumulative dose optimisation for lung cancer reirradiation within a commercial TPS. A patient- and organ- specific robustness strategy incorporating both DIR and radiobiological (α/β) uncertainty was implemented, to inform OAR specific resampling kernels. Equieffective plans were broadly superior to point dose maximum based planning and showed similar robustness to deformation and radiobiology uncertainty.

Blinded evaluation suggested that for patients with substantial irradiated volume overlap, our approach improved optimisation over current manual methods, especially when tumours share the same superior-inferior plane. Our manual comparator did not employ avoidance substructures or similar strategies used clinically in some centres to improve coverage, which can narrow the apparent improvement in plan quality but remain labour-intensive and registration-dependent compared with STRIDeR [2].

The proposed approach is less laborious, increases information for planners, and integrates into standard processes with few manual calculations, lowering transcription risk. Across six cases, the manual method required deriving and independently checking eight patient-specific combined clinical goals per case (48 calculations and 48 checks), whereas the automated pathway obviated these, leaving only clinical review.

We introduced equieffective cumulative dose evaluation directly into plan optimisation, evaluated it in a lung cohort, and compared qualitatively and quantitatively with manual approaches. This extended Murray *et al.* [7], who described STRIDeR in pelvic patients. Alternative optimisation methods have avoided dedicated reirradiation-specific cost functions [18]. McVicar *et al.* [14] provided a proof-of-concept for a dedicated reirradiation planning approach in lung cancer, using a ‘base plan’ with per-voxel dose determined by OAR constraints and the reirradiation fractionation scheme. Meyer *et al.* demonstrated auto-generated optimisation structures from isodose curves of robustly mapped previous doses [19]. Although this method integrates into any clinical TPS, it lacks flexibility in optimisation functions and objectives. Other ‘workaround’ approaches have been proposed, but they do not fully account for the cumulative equieffective dose in the plan optimisation [19]. Whilst the STRIDeR approach may initially challenge planners familiar with the manual approach or isodose curve-based methods, we consider it reasonably intuitive, even for less experienced planners. It builds on familiar optimisation objectives, and automated or templated planning can easily be integrated.

Reirradiation planning based on previous 3D distributions is sensitive to registration and equieffective dose uncertainties [20]. Over-optimisation with STRIDeR was rare in this cohort; more robust planning may have helped in two cases. The small cohort limits generalisability, and larger studies may demonstrate less robustness in ‘extreme’ cases. We did not incorporate 4D anatomical effects, and breathing-related uncertainties may require non-spherical resampling kernels. It may also be desirable to set a minimum resampling radius that encompasses neighbouring voxels, as sufficiently small MDA will have a sub-voxel resampling kernel. Volumetric constraints were not considered.

The dominant source of uncertainty varied by organ. For the brachial plexus and pericardium, geometric (dose-mapping) uncertainty was more influential, whereas for others α/β assumptions predominated. Both sources must be considered: although radiobiological uncertainty often dominates, geometric uncertainty can be clinically more significant for specific structures.

In conclusion, this novel optimisation approach may increase target dose whilst keeping OAR doses within tolerances. Larger retrospective studies are needed to confirm applicability in the lung, especially with greater anatomical differences and other fractionation regimes.

## CRediT authorship contribution statement

**David P. Walton:** Conceptualization, Data curation, Formal analysis, Investigation, Methodology, Visualization, Writing – original draft, Writing – review & editing. **Christopher Thompson:** Conceptualization, Supervision, Visualization, Writing – review & editing. **Dominic Lowe:** Data curation, Writing – review & editing. **Christopher J.H. Pagett:** Data curation, Writing – review & editing. **John Lilley:** Conceptualization, Resources, Writing – review & editing. **Stina Svensson:** Methodology, Resources, Software, Writing – review & editing. **Kjell Eriksson:** Software, Writing – review & editing. **Rasmus Bokrantz:** Software, Writing – review & editing. **Jakob Ödén:** Software, Writing – review & editing. **Louise Murray:** Conceptualization, Writing – review & editing. **Mark Teo:** Conceptualization, Data Curation, Writing – review & editing. **Ane Appelt:** Conceptualization, Methodology, Supervision, Writing – review & editing.

## Declaration of competing interest

The authors declare the following financial interests/personal relationships which may be considered as potential competing interests: Stina Svensson, Kjell Eriksson, Rasmus Bokrantz, and Jakob Ödén are full-time employees of RaySearch Laboratories AB, where Kjell Eriksson is also a shareholder. Leeds Teaching Hospitals NHS Trust holds a formal research collaboration agreement with RaySearch Laboratories.
