# Supplementary material for: Treatment planning for lung cancer reirradiation accounting for previously delivered dose
Source: Phys Imaging Radiat Oncol. 2026 Feb 6;38:100912. doi: 10.1016/j.phro.2026.100912 (PMC12993372; doi:10.1016/j.phro.2026.100912)
Supplement: Supplementary Data 1 [file mmc1.pdf]

Supplementary Materials

Table S1: Patient and treatment planning target volume (PTV) characteristics

Patient		1	2	3	4	5	6
Sex		Female	Male	Female	Male	Male	Female
Age		68	61	90	54	78	78
Previous Treatment	Dose and Fractionation	60 Gy in 8 #	55 Gy in 5 #	55 Gy in 5 #	55 Gy in 5 #	50 Gy in 15 #	55 Gy in 5 #
	Location	Left upper lobe	Left lower lobe	Right upper lobe	Right upper lobe	Right upper lobe	Right upper lobe
	Staging	T2a N0 M0	T1a N0 M0	T1b N0 M0	T2a N0 M0	T2a N0 M0	T1a N0 M0
	PTV volume (cm3)	36.7	17.9	49.0	71.5	146.7	22.7
Interval between courses (months)		8	21	9	9	19	23
Re-Irradiation	Location	Left lower lobe	Main tumour- right upper lobe	Nodal station R4	Right mediastinal nodes	Right upper lobe	Right hilar nodes
			Left hilar modes, nodal station L5				
	Site of Re-Irradiation	Localised regional progression	New Primary	Nodal recurrence	Nodal recurrence	Nodal recurrence	Nodal recurrence
			Local recurrence				
	PTV volume (cm^3)	275.8	256.6	56.7	119.9	123.4	33.9
PTV overlap		No	No	Touching	No	Yes	No
Same superior-inferior plane		Touching	Yes	Yes	Yes	Yes	No

Supplementary Material A: Planning Information

Original Plans (delivered clinically)

All patients had original radiotherapy treatments planned with an ITV based on tumour motion assessed on 4DCT and an additional 5 mm margin to the PTV. They received daily image guided radiotherapy. They were immobilised with a wingboard and a VacLok bag, head-first supine. The original treatment plan was made in Monaco (Monaco TPS (Elekta AB, Sweden)) using a Monte Carlo algorithm for dose calculation.

Reirradiation Plans

We planned all treatments on 2 mm slice thickness thoracic CTs, with PTV margins based either on 4D CT motion information or standard institutional margin protocols using 3D CT data.

Re-irradiation plans were made in RayStation11A (RaySearch Medical Laboratories AB, Sweden) using volumetric modulated arc therapy (VMAT) with a sliding window multi-leaf collimator technique with 6 MV flattening filter free energy. Right-sided PTV were planned with a partial arc 180° to 20° clockwise and left-sided PTV were planned 340° to 179.9° clockwise. Patient 2 had PTV in both lungs and was planned with two beams; one was 0° to 179.9° clockwise, the second, 180° to 0° clockwise. The gantry spacing was 2°. The TPS algorithm used was RayStation's Collapsed Cone. Single beam treatments had a maximum delivery time of 90 s. The two-beam treatment had 50 s per beam. There was no maximum monitor unit limit.

Table S2: Optimal and mandatory clinical goals used for the study for organs at risk (OAR), planning target volume (PTV) and clinical target volume (CTV). The Spinal Canal planning organ at risk volume (PRV) is 5 mm. Dose metrics evaluated on the reirradiation (reRT) treatment plans represent physical dose, otherwise all are in equieffective dose in 2 Gy per fraction (EQD2Gy) and represent the combined dose from both treatments. The Leeds Teaching Hospitals NHS Trust (LTHT) clinical protocol (CP) was used for the reirradiation only plan; references for remaining clinical goals are provided below. α/β values are those used for primary dose evaluation, with numbers in brackets representing the range used for plan robustness evaluation.

Parameter	α/β [Range used for robustness]	Dose metric [units]	Evaluated for	Optimal	Mandatory	Reference
PTV		$D_{99\%}$ [Gy]	ReRT dose	-	92% of $D_{prescription}$	LTHT CP
		$D_{2\%}$ [Gy]	ReRT dose	105% of $D_{prescription}$	110% of $D_{prescription}$	LTHT CP
		$D_{50\%}$ [Gy]	ReRT dose	within 2% of $D_{prescription}$	within 2% of $D_{prescription}$	LTHT CP
CTV		$D_{99\%}$ [Gy]	ReRT dose	-	95% of $D_{prescription}$	LTHT CP
		$D_{50\%}$ [Gy]	ReRT dose	within 2% of $D_{prescription}$	within 2% of $D_{prescription}$	LTHT CP
Lungs		D_{mean} [Gy]	ReRT dose	15.0	18.0	LTHT CP
		$V_{20\ Gy}$ [%]	ReRT dose	-	30	LTHT CP
Contralateral Lung		$V_{20\ Gy}$ [%]	ReRT dose	10.0	-	LTHT CP
		$V_{10\ Gy}$ [%]	ReRT dose	50.0	-	LTHT CP
		$V_{5\ Gy}$ [%]	ReRT dose	70.0	-	LTHT CP
		D_{mean} [Gy]	ReRT dose	8.0	-	LTHT CP
Spinal Canal	1 [0.75, 1.75, 2.75]	$D_{0.1\ cm^3}$ [EQD2Gy]	Combined dose	-	60.0	[S1,S2]
		$D_{0.1\ cm^3}$ [Gy]	ReRT dose	-	50.0	LTHT CP
Spinal Canal PRV		$D_{0.1\ cm^3}$ [Gy]	ReRT dose	-	54.0	LTHT CP
Pericardium	2.5 [2, 3, 4, 5, 6]	$D_{0.1\ cm^3}$ [EQD2Gy]	Combined dose	ALARA	85.0	[S3]
		D_{mean} [EQD2Gy]	Combined dose	ALARA	70.0	[S4]
Oesophagus	3 [2, 3, 4, 5, 6]	$D_{1.0\ cm^3}$ [EQD2Gy]	Combined dose	75.0	100.0	[S1]
Trachea	3 [2, 3, 4, 5, 6]	$D_{0.1\ cm^3}$ [EQD2Gy]	Combined dose	80.0	105.0	[S1]
Proximal Bronchial Tree	3 [2, 3, 4, 5, 6]	$D_{0.1\ cm^3}$ [EQD2Gy]	Combined dose	80.0	105.0	[S1]
Brachial Plexus	2 [1, 2, 3, 4, 5, 6]	$D_{0.1\ cm^3}$ [EQD2Gy]	Combined dose	80.0	95.0	[S1]
		$D_{0.1\ cm^3}$ [Gy]	ReRT dose	-	66.0	LTHT CP
Vessels	3 [2, 3, 4, 5, 6]	$D_{0.1\ cm^3}$ [EQD2Gy]	Combined dose	110.0	115.0	[S1]

Table S3: Mean distance to agreement (MDA) for organs at risk (OAR) contours mapped from previous CT onto reirradiation CT relative to OAR contours outlined directly on the reirradiation CT.

Organs at risk	Patient 1 (mm)	Patient 2 (mm)	Patient 3 (mm)	Patient 4 (mm)	Patient 5 (mm)	Patient 6 (mm)
Proximal Bronchial Tree	0.8	1.7	1.1	1.5	2.0	0.7
Brachial Plexus	5.1	4.1	6.3	5.3	4.7	2.1
Oesophagus	1.7	1.9	1.8	2.0	1.7	1.5
Pericardium	1.6	2.5	2.0	2.1	2.0	2.0
Spinal Canal	0.8	0.9	1.0	0.8	1.1	1.0
Trachea	0.8	1.9	2.5	2.2	2.0	1.9
Vessels	0.8	1.9	1.8	1.8	1.8	2.1

Table S4: **Robust evaluation of organ-at-risk dose-volume metrics against combined clinical goals**

The combined clinical goals (EQD2Gy) for each organ-at-risk are shown in the first column. For each patient uncertainties from both image registration and the α/β ratio were used to generate a robust, worst-case scenario dose-volume metric for the STRIDeR and manual plans. These robust metrics were then compared with the clinical goals to determine whether each plan would still meet the goal under the combined uncertainty.

**Robustness**			**1**		**2**		**3**		**4**		**5**		**6**	
**Quantitative (EQD2Gy)**	Combined Clinical Goals		STRIDeR	Manual	STRIDeR	Manual	STRIDeR	Manual	STRIDeR	Manual	STRIDeR	Manual	STRIDeR	Manual
**Mandatory**	Proximal Bronchial Tree ($D_{0.1\ cm^3}$)	105	63.7	63.1	51.0	41.5	65.9	63.8	82.4	83.0	106.8	102.6	63.0	62.6
	Brachial Plexus ($D_{0.1\ cm^3}$)	95	116.8	116.8	1.6	1.7	109.2	109.9	101.3	101.3	0.7	0.7	54.7	54.7
	Oesophagus ($D_{1.0\ cm^3}$)	100	61.3	58.7	23.1	23.4	59.6	68.3	59.6	60.1	66.8	66.1	26.4	26.4
	Pericardium ($D_{0.1\ cm^3}$)	85	63.6	63.4	81.1	59.3	75.5	64.8	73.1	74.6	84.8	79.3	63.8	62.0
	Pericardium (D_{mean})	70	6.7	6.8	13.2	11.1	4.0	3.7	4.1	4.2	12.4	10.5	2.1	2.1
	Spinal Canal ($D_{0.1\ cm^3}$)	60	37.0	37.2	18.6	14.4	14.9	17.0	20.2	22.2	38.3	30.5	11.1	11.9
	Trachea ($D_{0.1\ cm^3}$)	105	62.4	59.0	16.2	18.9	103.9	106.7	78.7	78.2	68.7	67.9	36.8	36.8
	Great Vessels ($D_{0.1\ cm^3}$)	115	79.7	79.5	63.6	64.7	105.2	103.5	95.6	95.9	100.5	71.2	63.0	61.6
**Optimal**	Proximal Bronchial	80	63.7	63.1	51.0	41.5	65.9	63.8	82.4	83.0	106.8	102.6	63.0	62.6

	Tree ($D_{0.1\ cm^3}$)												
	Brachial Plexus (D 0.1 cm3)	80	116.8	116.8	1.6	1.7	109.2	109.9	101.3	101.3	0.7	0.7	54.7	54.7
	Oesophagus ($D_{0.1\ cm^3}$)	75	61.3	58.7	23.1	23.4	59.6	68.3	59.6	60.1	66.8	66.1	26.4	26.4
	Trachea ($D_{0.1\ cm^3}$)	80	62.4	59.0	16.2	18.9	103.9	106.7	78.7	78.2	68.7	67.9	36.8	36.8
	Great Vessels ($D_{0.1\ cm^3}$)	110	79.7	79.5	63.6	64.7	105.2	103.5	95.6	95.9	100.5	71.2	63.0	61.6

Table S5: A breakdown was made of the contributions from α/β ratio uncertainty and geometric uncertainty. Separate robust sums were calculated for each source. For six patients, the difference in near-maximum point doses between robust and standard sums was recorded for each organ with combined clinical goals. The median and range were calculated. This was performed for both the manual plan and the STRIDeR plan, allowing identification of which uncertainty contributed more to each organ at risk.

	Median Radiobiology Uncertainty (Gy)		Median Geometric Uncertainty (Gy)		Which uncertainty Dominates? (average difference)
	Manual	SRIDeR	Manual	SRIDeR	
Proximal Bronchial Tree ($D_{0.1\ cm^3}$)	0.6 (0.1–1.9)	0.7 (0.1–2.2)	0.0 (0.0–2.0)	0.1 (0.0–2.3)	Radiobiology Uncertainty (0.6 Gy)
Brachial Plexus ($D_{0.1\ cm^3}$)	6.0 (0.1–9.4)	4.7 (0.1–9.5)	16.7 (0.0–29.2)	15.3 (0.0–29.2)	Geometric Uncertainty (10.7 Gy)
Oesophagus ($D_{1.0\ cm^3}$)	1.1 (0.4–2.3)	1.1 (0.0–2.3)	0.6 (0.0–1.3)	0.6 (0.0–1.3)	Radiobiology Uncertainty (0.5 Gy)
Pericardium ($D_{0.1\ cm^3}$)	0.4 (0.0–1.4)	0.2 (0.1–0.9)	0.9 (0.1–4.0)	1.0 (0.1–5.1)	Geometric Uncertainty (0.6 Gy)
Spinal Canal ($D_{0.1\ cm^3}$)	1.8 (1.6–2.4)	1.8 (1.4–3.0)	0.0 (0.0–0.0)	0.0 (0.0–0.0)	Radiobiology Uncertainty (1.8 Gy)
Trachea ($D_{0.1\ cm^3}$)	1.4 (0.3–3.3)	1.3 (0.0–3.0)	0.4 (0.0–4.0)	0.4 (0.0–3.3)	Radiobiology Uncertainty (0.9 Gy)
Great Vessels ($D_{0.1\ cm^3}$)	0.4 (0.1–2.9)	0.5 (0.1–3.6)	0.0 (0.0–1.7)	0.0 (0.0–1.6)	Radiobiology Uncertainty (0.5 Gy)

References

[S1] Rulach R, Ball D, Chua KL, Dahele M, De Ruysscher D, Franks K, et al. An international expert survey on the indications and practice of radical thoracic reirradiation for non-small cell lung cancer. Adv Radiat Oncol 2021;6:100653. https://doi.org/10.1016/j.adro.2021.100653.

[S2] Kirkpatrick JP, Van der Kogel AJ, Schultheiss TE. Radiation dose–volume effects in the spinal cord. Int J Radiat Oncol Biol Phys 2010;76:S42–S49. https://doi.org/10.1016/j.ijrobp.2009.04.095.

[S3] Paradis KC, Mayo C, Owen D, Spratt DE, Hearn J, Rosen B, et al. The special medical physics consult process for reirradiation patients. Adv Radiat Oncol 2019;4:559–565. https://doi.org/10.1016/j.adro.2019.05.007.

[S4] Troost EGC, Wink KCJ, Roelofs E, Simone CB 2nd, Makocki S, Löck S, et al. Photons or protons for reirradiation in (non-)small cell lung cancer: results of the multicentric ROCOCO in silico study. Br J Radiol 2020;93:20190879. https://doi.org/10.1259/bjr.20190879.
